# Single-Stage Revision Surgery in Infected Total Knee Arthroplasty: A PRISMA Systematic Review

**DOI:** 10.3390/jcm8020174

**Published:** 2019-02-02

**Authors:** Khaled M. Yaghmour, Emanuele Chisari, Wasim S. Khan

**Affiliations:** 1 Division of Trauma & Orthopaedic Surgery, University of Cambridge, Addenbrooke’s Hospital, Cambridge CB2 0QQ, UK; khalid.yagh@gmail.com (K.M.Y.); chisari.emanuele@gmail.com (E.C.); 2 Department of General Surgery and Medical Surgical Specialties, Section of Orthopaedics and Traumatology, University Hospital Policlinico, University of Catania, 95123 Catania, Italy

**Keywords:** single-stage revision, infected total knee arthroplasty, periprosthetic joint infection, reinfection rate, functional outcome

## Abstract

Periprosthetic joint infection in total knee arthroplasty is a significant complication that is a common reason for revision surgery. The current standard of care is two-stage revision surgery. There is however increasing evidence to support the use of single-stage revision surgery. We conducted a PRISMA systematic review of the current evidence on the use of single-stage revision for infected total knee arthroplasty. Four databases (PubMed, Embase, Science Direct, and Cochrane Library) were systematically screened for eligible studies. The risk bias of each study was identified using ROBINS-I tool, and the quality of evidence was assessed using the GRADE criteria. Sixteen articles were retained after applying the inclusion and exclusion criteria that evaluated 3645 knee single-stage revision surgeries. Our review reveals satisfactory outcomes for single-stage revision in the management of infected total knee arthroplasty. The reinfection rates in the studies included in our review varied however the majority reported low reinfection rates and good functional outcomes. Although strict patient selection criteria have yielded successful results, good results were also reported when these criteria were not applied. The greater use of risk factors in identifying patients likely to have a successful outcome needs to be balanced with the practical benefits of performing a single stage procedure in higher risk patients. Future large clinical randomized control trials are required to confirm our results.

## 1. Introduction

According to the United Kingdom (UK) National Joint Registry (NJR), there have been 1,087,611 total knee arthroplasty (TKA) procedures reported from the 1st of April 2003 till the 31st of December 2017 [1]. The most common indication for surgery was osteoarthritis, accounting for 97% of the cases [1]. Women were more commonly operated on than men, and the mean age at the time of operation was 68.9 years [1]. In the United States (US) alone it is approximated that the need for TKA will substantially increase by 673% equating to 3.48 million procedures by 2030 [2]. Periprosthetic joint infection (PJI) is one of the most dreaded complications of TKA, and is a well-defined reason for revision surgery [3,4]. In a recent study [5], it was concluded that PJI account for 26.8% of revision surgeries. It is approximated that by 2030 $13 billion will be spent annually by the US health system on revision surgeries [2]. Several factors are associated with an increased risk of developing PJI, these include a surgical site infection not involving the joint prosthesis, a National Nosocomial Infections Surveillance (NNIS) System surgical patient risk index score of 1 or 2 and existing malignancy [6].

Various surgical options are available for the management of PJI after a TKA. These include debridement and retention of the prosthetic implants, two-stage exchange revision, single-stage or direct exchange revision, permanent resection arthroplasty, and finally amputation as the last measure [7]. Although debridement and retention of the prosthetic implants is a viable option in the early stages of an acute infection, established infection does necessitate the removal of all prostheses. A large volume of literature reports good outcomes with two-stage revision of with a clearance rate of 90% [8]. The fact that patients undergo two surgeries in two-stage revision increases the chance of eradicating the infection, and provides sufficient time to evaluate the response of the patient to the antibiotics before definitive implants are introduced at the second stage [9]. However, this restricts the patient’s daily activities between the two surgeries when the patient will have a temporary implant, carries the risk of complications associated with the temporary implant and of the two anesthetics, and is associated with a high financial cost [9].

Recently single-stage revision surgery has gained increased popularity in the management of PJIs; it includes only one surgical procedure, and is linked to better outcomes in terms of morbidity, mortality, cost and total duration of the procedure [4,10]. Studies investigating single-stage revision show promising results. The reinfection rate (RR) of single-stage revision surgery (5–25%) [8,11,12,13,14,15,16,17,18] is comparable to two-stage revision (9–20%) [3]. In particular, in a study of 28 highly selected patients undergoing single-stage revision surgery for chronically infected knee arthroplasties, it was shown that patients were free of reinfection after a 3 year follow-up [8]. In another study investigating 50 knee revision surgeries with an average follow-up of 10.5 years, the reinfection rate was reported to be 2% [19]. The evidence supporting single or two-stage approach for PJI is not definitive. The aim of this review is to report the most up-to-date evidence on single-stage revision for infected TKA.

## 2. Methods

### 2.1. Literature Search Strategy

This systematic review was conducted according to the guidelines of the Preferred Reporting Items for Systematic Reviews and Meta-Analyses (PRISMA) [20]. A comprehensive search was performed on four medical electronic databases (PubMed, Embase Science Direct, and Cochrane Library) by two independent authors (K.M.Y. and E.C.) from the 1st of January 1998 to the 20th of October 2018. Our main aims were to: (1) determine the reinfection rate after single-stage revision, (2) identify risk factors associated with the main outcome, and (3) evaluate functional outcome through validated scores. To achieve the maximum sensitivity of the search strategy, we combined the terms: ‘‘knee” with “(replacement OR arthroplasty)”, ‘‘knee” with “(single-stage exchange) OR (single-stage revision)” as either key words or MeSH terms. The reference lists of all included articles, previous literature reviews on the topic and top hits from Google Scholar were reviewed for further identification of potentially relevant studies and were assessed using the inclusion and exclusion criteria. In order to avoid overlap with other ongoing reviews, we first searched the PROSPERO site for any similar review and then prospectively registered our study (registration number: CRD42018116838).

### 2.2. Selection Criteria

Eligible studies for the present systematic review included those investigating the use of single-stage surgery for the revision of infected TKA. Primary screening of the titles and abstracts was performed and all studies of any level of evidence published in peer-reviewed journals reporting clinical results were included. Moreover, articles discussing both single-stage and two-stages were reviewed. Exclusion criteria included studies investigating revision surgeries for non-infective reasons such as aseptic loosening, periprosthetic fractures and persistent pain. Additionally, we excluded studies in which data about single-stage revision was not accessible, missing, without an available full text, or not well reported. We also excluded all duplicates, and studies with poor scientific methodology. All publications were limited to in vivo human studies in the English language. Abstracts, case reports, conference presentations, reviews, editorials and expert opinions were excluded. The study selection was performed independently by two authors (K.M.Y. and E.C.), and any discrepancies in the selection process were resolved by discussion amongst the authors. A senior investigator (W.S.K.) was consulted to revise the selection process.

### 2.3. Data Extraction and Criteria Appraisal

All data was extracted from article texts, tables and figures. Data was extracted using the Population, Intervention, Comparison, Outcome (PICO) framework and included title, year of publication, study design, sample size, study population, patient characteristics, intervention and comparator (where applicable), outcomes, funding and conclusions. Two investigators independently reviewed each article (K.M.Y. and E.C.). Discrepancies between the two reviewers were resolved by discussion and consensus. The final results were reviewed by the senior investigator (W.S.K.).

### 2.4. Risk of Bias Assessment

Risk of bias assessment of all in vivo selected full-text articles was performed according to the ROBINS-I tool for non-randomized studies [21] (Supplementary Materials Table S1). This assessment used “Low,” “Moderate,” and “High” as judgement keys: “Low” indicated a low risk of bias, “moderate” indicated that the risk of bias was moderate, and “High” indicated a high risk of bias. The assessment was performed by two authors (K.M.Y. and E.C.) independently. Inter-rater agreement was 92%. Any discrepancy was discussed with the senior investigator (W.S.K.) for the final decision.

### 2.5. Study Quality Assessment

The quality of evidence and risk of bias were assessed using the Grading Recommendations Assessment and Development Evidence (GRADE) criteria with supporting guidance from the Cochrane website [22]. The GRADE approach considers five elements, namely, risk of bias, imprecision, inconsistency, indirectness, and publication bias, for downgrading the overall quality of evidence. We presented the main findings of the review in a tabular format (Supplementary Materials Table S2). Results are reported as “Very low”: the true effect is probably markedly different from the estimated effect, “Low”: the true effect might be markedly different from the estimated effect, “Moderate”: the authors believe that the true effect is probably close to the estimated effect, and “High”: the authors have a lot of confidence that the true effect is similar to the estimated effect. The assessment was performed by two authors (K.M.Y. and E.C.) independently. The inter-rater agreement was 94%. Any discrepancy was discussed with the senior investigator (W.S.K.) for the final decision.

### 2.6. Statistical Methods and Analysis

All statistical analyses were performed with SPSS software (version 17.0; IBM, Armonk, NY, USA). Quantitative variables were presented as mean and standard deviation. We analyzed the correlation between the quantitative variables extracted from the included studies and the reinfection rate. In order to assess the influence of the quality of the studies included on the reinfection rate outcome we used the Welch independent sample t test because of the unequal distribution of the samples. Differences with *p* < 0.05 were considered statistically significant. Due to the heterogenous nature of the studies and the lack of controlled studies, it was not possible to perform a metanalysis and descriptive synthesis was undertaken.

## 3. Results

### 3.1. Study Selection

**Figure 1:**
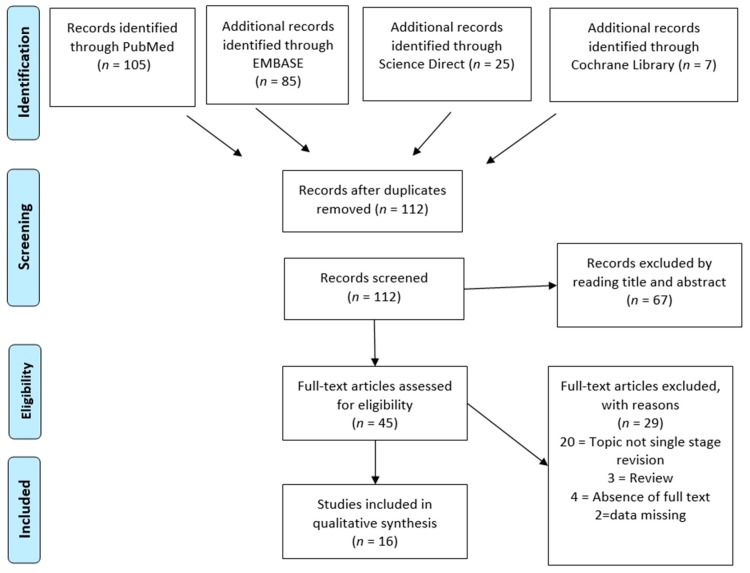
PRISMA flowchart of the systematic literature review.

A total of *n* = 222 studies was collected from the databases using the aforementioned inclusion and exclusion criteria. Overall *n* = 112 papers were screened through abstract and title reading after the removal of the duplicates. Ultimately, after reading the full texts and checking the reference lists, we selected *n* = 16 articles for analyses in our systematic review. A PRISMA [20] flow chart of the selection process and screening is provided in Figure 1.

### 3.2. Study Characteristics

Of the sixteen included studies [8,11,12,13,14,15,16,17,18,19,21,22,23,24,25,26] that met the inclusion criteria, thirteen were retrospective studies [8,11,12,13,14,15,16,17,21,22,23,24,25]; nine were retrospective cohort studies [8,11,12,15,17,22,23,24,25], one was a retrospective case series [21], one was a retrospective descriptive study [16], one was a case-control study [13], and one was a large retrospective observational database study [14]. Three were prospective studies [18,19,26]; two prospective cohort studies [19,26], and one prospective observational cohort study [18]. Overall, 3,645 single-stage knee revision surgeries were included in this systematic review. The study design, patient demographics, follow-up, outcomes, and main findings are summarized in Table 1, and the details of the surgical techniques used in each study are included in Table 2.

In our review the reinfection rate ranged from 0–38%, with a mean of 15.42% (Median 15.00%, SD ± 10.42). We further evaluated the variability of reinfection rate between high and moderate quality studies. We found a significant (*p* = 0.003) association between lower reinfection rates and studies assessed as “high quality” (t: –3.551; df: 13.83). The scoring systems used in our included studies were Hospital for Special Surgery [HSS] score, International Knee Society Score [IKS], Knee society Score [KSS], Oxford–12 Knee Score [OKS], and Western Ontario and McMaster Universities Osteoarthritis Index [WOMAC] function score. The most commonly used function scores were the KSS (38.5%), followed by OKS (23%) and HSS (23%). In the high-quality studies, the most commonly utilized function scores were KSS followed by OKS, whereas the most commonly used function scores in the moderate quality studies were KSS and HSS.

## 4. Discussion

Several surgical techniques are available for the management of established PJI in knee arthroplasty and these commonly include a single or two–stage revision. Controversy however exists regarding the use of single–stage revision surgery as some studies in the literature report a higher reinfection rate [3,8,11,12,13,14,15,16,17,18,27]. In this study, we systematically reviewed the available evidence regarding the effectiveness of single–stage revision in infected TKA with a focus on reinfection rate and functional outcome.

### 4.1. Reinfection Rates

Kunutsor et al. [28] looked at aggregate published data looking at reinfection rate within two years of revision for single–stage and two–stage revision knee surgery. They found both approaches to be effective in treating infected knee prostheses in generally unselected patients with reinfection rates of 7.6% (3.4–13.1%) in single–stage studies and 8.8% (7.2–10.6%) in two–stage revision. The largest study [14] reporting on single–stage revision surgery included in our review investigated 3069 patients through the Medicare registry in the United States. The reinfection rate for single–stage revision surgery was reported to be 24.6% and 38.25% at 1 year and 6 years respectively. This was higher than the reinfection rate for two–stage revision surgery reported as 19% and 29% at 1 year and 6 years respectively. The Medicare registry is a large national database in the United States and invariably includes a heterogenous mix of patients with a wide range of risk factors. This highlights the regional variation in surgical practices and outcomes.

**Figure 2:**
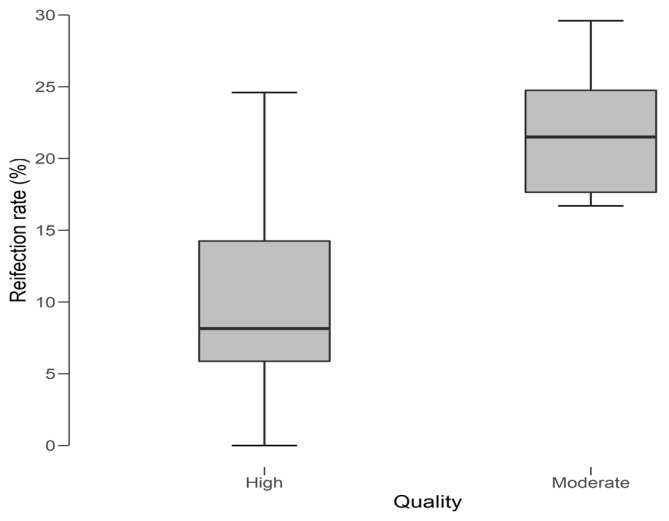
Reinfection rate (%) reported against the quality of the included studies.

**Figure 3:**
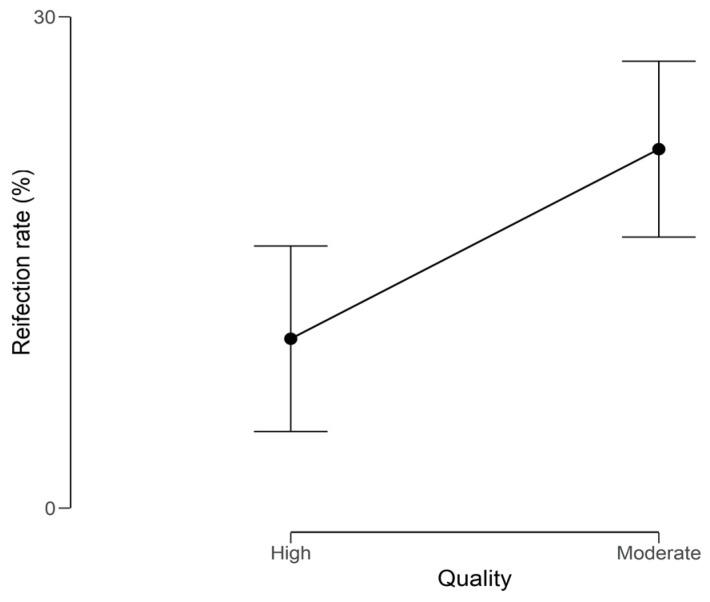
Welch t–student test distribution plot of reinfection rate (%) against the quality of the included studies.

When analyzing the reinfection rate (%) against the quality of our included studies (Figure 2) we observed a lower, yet wider range of reinfection rates in the high quality studies when compared to the moderate quality studies. This translated into a significant difference (*P* = 0.003) in reinfection rate outcome between the high and moderate quality studies (Figure 3).

This difference could be explained by the stronger methodology standards utilized in the studies assessed as “high quality” compared to the studies assessed as “moderate quality”. A well–structured methodology plays a substantial role in producing more accurate and reliable result, which leads to a stronger conclusion that is clinically relevant. In addition, we found that 50% of the moderate quality studies did not describe their surgical procedure used in the single–stage revision. In addition, our review did not reveal any significant difference in the age of the patients, and follow–up duration between the high and moderate quality studies that could account for the considerable differences noted in the reinfection rates.

### 4.2. Risk Factors for Reinfection

Although a high reinfection rate of 23% was noted by Massin et al. [15] in a population with an average age of 71 years, Kunutsor et al. [28] and Cochran et al. [14] reported no significant association between age and developing reinfection after revision of infected total knee arthroplasty.

High reinfection rates have been associated with obstructive sleep apnea (OSA) and body mass index (BMI) > 30 kg/m^2^ [11,13]. Although Cochran et al. [14] did not report on the BMI status of the patients due to concerns over completeness and accuracy of data, a high BMI is another significant risk factor in developing reinfections owing to the prolonged procedure duration, comorbidities, and poor delivery of the antibiotic to the surrounding tissues [29,30,31].

Previous joint surgeries was also reported as risk factor for developing recurrent infections in a number of studies [28,32,33]. In a study [16] comparing recurrence of infection between primary total knee arthroplasty prosthesis and rotating hinged prosthesis, it was shown that rate of infection recurrence was 0% and 15% respectively. None of the primary total knee arthroplasty prosthesis group had previous surgery whereas 30% of the hinge prostheses patients had previous septic revisions. Also, 35% of the group with the hinge prosthesis had a fistula compared to 5.4% in the primary total knee arthroplasty prosthesis. Klatte et al. [24] reported one case of revision for recurrent infection out of 4 patients with hinged prostheses; the recurrent infection was immunocompromised due to prolonged steroid use and has diabetes.

Other risk factors associated with developing reinfection after a single–stage revision surgery include a history of diabetes, rheumatoid arthritis, smoking, depression and previous steroid use [28]. In a study [19] investigating single–stage revision surgery on 50 knees it was concluded that rheumatoid arthritis patients have a twenty–fold higher odds of death than osteoarthritis (*p* = 0.04). Massin et al. [15] identified other factors associated with infection recurrence including fistulae, infection by gram–negative bacteria, and two–stage surgery with static spacers. Although *Staphylococcus aureus* is a common organism in PJI [8,14,22], failure rates in single–stage revision surgeries are considerably higher in patients with fungal infections despite following a strict antifungal and antibiotic regimen [21,24].

### 4.3. Functional Outcomes

Although all of the included studies in our review reported successful outcomes following single–stage revision [8,13,15,16,17,18,19,21,22,23,24,25,26], heterogeneity of the reported functional outcome scores meant that it was not possible to perform a meaningful statistical comparison. Singer et al. [16] concluded that the KSS postoperatively in single–stage revision was higher than that reported for two–stage revision. Additionally, the KSS was significantly (*p* < 0.02) higher in single–stage revision surgery patients compared to two–stage revision patients as reported by Haddad et al. [8]. Ji et al. [21] showed a significant improvement in HSS score postoperatively in patients managed for fungus infected knee prosthesis. Klatte et al. [24] also noted the mean HSS knee score to significantly increase from 51 to 75. Significant postoperative improvements in OKS were reported by three studies [16,19,26].

### 4.4. Patient Selection Criteria

Recent evidence suggests that single–stage revision surgery is a viable option in the management of infected TKA [8,11,12,16,18,19,25,27]. Surgeon preference and strict patient selection criteria can influence the rate of success of single–stage revision surgery [8,11,18,21]. Haddad et al. [8] and Li et al. [12] reported a reinfection rate of 0% in 28 PJIs and 9.09% in 22 PJIs respectively when applying a strict selection protocol. Li et al. [12] reported a success rate of 90.9% in patients undergoing single–stage revision surgery, and attributed the results to the fact that their patients underwent the procedure only if there was a positive response to the antibiotic treatment. In addition, antibiotics were commenced in accordance to the drug sensitivity test, which allows for more specified microorganism targeting and decreases the chance of antibiotic resistance. Jenny et al. [13] investigated 131 patients that underwent single–stage revision surgery and found no significant difference in reinfection rates when comparing a study group (*n* = 54) with no strict selection criteria with a control group (*n* = 77) with a strict selection criteria. Zahar et al. [23] investigated 46 patients and concluded that the 10–year infection–free survival rate was 93% despite not adhering to a strict selection criteria protocol, however in this study the specific patient demographics and comorbidities were not reported. These conflicting results highlight the need for more work to be done to better define the role of selection criteria in deciding suitability for single–stage revision surgery.

Risk factors including immunosuppression, rheumatoid arthritis and malignancy are associated with increased perioperative complications, and warrant consideration [22,32,33]. Comorbidities increase the risk of reinfection, and patients with significant comorbidities have historically been managed with a two–stage revision. There is an argument for managing these patients with a single–stage procedure avoiding the need for two general anesthetics and the morbidities associated with an interim spacer between the two surgeries [32,34].

## 5. Limitations

The major limitation of this systematic review is the quality of the included studies. Most of the included studies are retrospective, with no clinical randomized controlled trials identified. Despite applying a specific time frame for our review, the management preference and infecting organisms have varied during this timeframe. We do however believe that this work summarizes and highlights the relevant evidence in the literature that would help guide clinical randomized controlled trials.

## 6. Conclusions

This study is the first PRISMA review of single-stage knee revision surgery for PJI to our knowledge. Our review reveals satisfactory outcomes for single-stage revision in the management of infected total knee arthroplasty. The reinfection rates in the studies included in our review varied however the majority reported low reinfection rates and good functional outcomes. Although strict patient selection criteria have yielded successful results, good results were also reported when these criteria were not applied. The greater use of risk factors in identifying patients likely to have a successful outcome needs to be balanced with the practical benefits of performing a single stage procedure in higher risk patients. Data from joint registries is useful but the heterogeneous nature of the cases makes interpretation difficult. Future good quality large clinical randomized control trials are needed to confirm our results and allow for more objective analysis of the surgical technique and success rate.

## Figures and Tables

**Table 1:** The main characteristics of the included studies.

Author	Study Design	Patient Demographics	Outcomes	Follow-up	Main Findings
Castellani et al. (2017)	Retrospective Cohort	*n* = 14 Mean age 68 (IQR 61–79) Gender: 19F, 16M	RR = 7.2% FO: Not reported	1 year	Superiority of single-versus two-stage revision and the value of antibiotic-free periods prior to definitive revision remain unclear. Large prospective studies or randomized controlled trials are needed to inform best practice for treatment of these complex clinical problems.
Ji et al. (2017)	Retrospective Case Series	*n* = 7 Mean age 66.5 ± 10.1 Gender: 5F, 2M	RR = 29.6% FO: HSS increased significantly from 46 points (38–57 points) preoperatively to 78 points (73–84 points; *p* < 0.05) postoperatively.	5 years	Treatment of chronic fungal periprosthetic joint infection with single–stage revision can be fairly effective for achieving acceptable functional outcomes, which indicated that this may be a feasible alternative strategy in selected patients
Li H et al. (2017)	Retrospective Cohort	*n* = 22 Mean age 64.4 ± 9.5 Gender: Not reported	RR = 9.1% FO: Not reported	5 years	The data reports no significant difference between single–stage and two–stage revision in terms of satisfaction rates, and overall infection control rates.
Massin et al. (2016)	Retrospective Cohort	*n* = 108 Mean age 71 (63–76) Gender: 55F, 53M	RR = 24% FO: IKS 88.6 ± 9.4	2 years	Results suggest that single–stage procedures are preferable, because they offer greater comfort without increasing the risk of recurrence. Routine single–stage procedures may be a reasonable option in the treatment of infected TKR.
Jenny et al. (2016)	Retrospective case–control	Intervention group = 54, Control group = 77, Mean age 70 (range, 45–90 years). Gender: 68F, 63M	RR = Intervention group: 15% Control group: 22% FO: KSS over 160 points (80%). No significant difference between the two groups	2 years	When single–stage exchange is considered, patient selection does not improve outcome.
Cochran et al. (2016)	Retrospective Observational Database	*n* = 3069 knees from Medicare records Gender: Not reported	RR = 24.6% at 1 year and 38.25% at 6 years FO: Not reported	6 years	Two–stage reimplantation, despite 19% recurrence, had the highest success rate. Given the higher failure rates of I&D and single–stage revisions, guidelines need to be established for their specific indications.
Zahar A et al. (2016)	Retrospective Cohort	*n* = 46 Mean age 70 (range, 60–81) Gender: Not reported	RR = 7% FO: HSS score improved significantly from a mean preoperative value of 35 (±24.2 SD; range, 13–99) to an average of 69.6 (±22.5 SD; range, 22–100)	10 years	The study results show an overall infection control rate of 93% and good clinical results using single–stage approach, which combines aggressive debridement of the collateral ligaments and posterior capsule with a rotating hinge implant.
Haddad et al. (2015)	Retrospective Cohort	*n* = 28 Mean age 65 years (range, 45–87 years) Gender: 14F, 14M	RR = 0% FO: KSS was higher in the single–stage group than in the two–stage group (mean, 88; range, 38–97 versus 76; range, 29–93; *p* < 0.001) Preoperative mean KSS was 32 in the single–stage group (range, 18–65)	2 years	The data provides preliminary support to the use of a single–stage approach in highly selected patients with chronically infected TKAs as an alternative to a two–stage procedure. However, larger, multicenter, prospective trials are called for to validate our findings.
Cury Rde P et al. (2015)	Retrospective Cohort	*n* = 6 Mean age 70.3 Gender: not reported.	RR = 16.7% FO: WOMAC score 49.5 (47–55)	3 years	The best results of quality of life (QoL) and function occur in patients undergoing debridement and retention. In contrast, the worst QoL and functional results were obtained in patients treated with two–stage revision arthroplasty.
Tibrewal et al. (2014)	Prospective Cohort	*n* = 50 Mean age 66.8 Gender: 33F, 17M	RR = 2% FO: the mean OKS increased by a factor of 2.4 from 14.5 (6 to 25) pre–operatively to 34.5 (26 to 38) one year after surgery. This represents a mean absolute improvement of 20.0 points (95% CI: 17.8 to 22.2, *p* < 0.001).	10. years	These results suggest that a single–stage revision can produce comparable results to a two–stage revision. Single–stage revision offers a reduction in costs as well as less morbidity and inconvenience for patients.
Klatte et al. (2014)	Retrospective Cohort	*n* = 4 Mean age 67.75 ± 13.3 Gender: 1F, 3M	RR = 25% FO: mean HSS knee score increased to 75 points (70 to 80; *p* < 0.01) from 51 points.	7 years	A single–stage revision following fungal periprosthetic infection is feasible, with an acceptable rate of a satisfactory outcome.
Shanmugasundaram et al. (2014)	Retrospective Cohort	*n* = 5 Mean age 63 ± 10 Gender: Not reported	RR = 17.2% FO: Not reported	2 years	Initial success of single–stage exchange was 80% and two–stage exchange 75%. Future advances in organism isolation and international standardization of treatment protocols may improve patient outcomes.
Baker et al. (2013)	Prospective Cohort	*n* = 33 Mean age 69.4 ± 10.7 Gender: 15F, 18M	RR = 21% FO: The mean pre– and post–operative OKS were 15 (95% CI, 13–18) and 25 (95% CI, 21–29), respectively, giving a mean improvement of 10 (95% CI, 5–14).	7 months	This study found no demonstrable benefit of single–stage compared to two–stage revision for the infected total knee arthroplasty using a variety of PROMs. Thus, the recommendation is that decision making must be based on other factors such as re–infection rate.
Jenny et al. (2013)	Observational Cohort Prospective	*n* = 47 Mean age 72 (range 45–93) Gender: 27F, 20M	RR = 12% FO: The median preoperative KSS function score was 42 points. 56% of the patients had a KSS of >150 points postoperatively	3 years	Single–stage exchange may be a reasonable alternative in chronically infected TKA as a more convenient approach for patients without the risks of two operations and hospitalizations and for reducing costs.
Singer et al. (2012)	Descriptive Retrospective	*n* = 57 Mean age 72 ± 8.7 Gender: 30F, 27M	RR = 15% FO: KSS after surgery was 72 points (range, 20–98 points), the Knee Society function score was 71 points (range, 10–100 points), and the Oxford–12 knee score was 27 points (range, 13–44 points).	3 years	Single–stage revision of septic knee prostheses achieved an infection control rate of 95% and higher knee scores than reported for two–stage revisions. Higher rates of recurrent infection appeared to be associated with long–term chronic infections of hinged prostheses
Whiteside et al. (2011)	Retrospective Cohort	*n* = 18 Mean age 69 ± 6 Gender 11F, 7M	RR = 5.5% FO: Mean KSS was 78 ± 8 at 1year, 83 ± 9 at 2 years, 84 ± 8 at 5years, 85 ± 10 at 6 years, and 84 at 8 years.	5.1 years	Single–stage revision and 6 weeks of intraarticular vancomycin administration–controlled infection in MRSA infected TKA with no apparent complications.

RR: reinfection rate, F: Female, M: Male, FO: functional outcome, HSS: Hospital for Special Surgery knee score, IKS: International Knee Society Score, KSS: Knee Society Score, OKS: Oxford Knee Score, WOMAC: Western Ontario and McMaster Universities Osteoarthritis Index, QoL: Quality of Life.

**Table 2:** Details of the surgical technique carried out in the included studies.

Author	Procedure Detail
Castellani et al. (2017)	Extensive irrigation and debridement, removal of components, immediately followed by re–implantation of new definitive components with a new set of sterile instruments under the same anesthetic.
Ji et al. (2017)	Aggressive debridement performed. 2nd aspiration done (1st was preoperatively). 3 samples acquired, components and cement debris removed. The surgical area was then irrigated with at least 2 L saline and 100–200 mL of 3% hydrogen peroxide and soaked in 400–500 mL of 0.1% aqueous Betadine for 15 min. The surgical area was resterilized and redraped, and the surgical team rescrubbed, and exchanged the entire set of surgical instruments. 0.5 g of dry vancomycin powder was poured into distal femoral and proximal tibia canal. Also, 0.5 g of gentamicin–loaded commercial cement was added. After that, another 0.5 g of vancomycin powder was poured into the whole joint cavity before closing the deep fascia. The wound was closed over a suction drain, which was retained for 3 days or if the volume of daily drainage was 50 mL.
Li H et al. (2017)	The infected prostheses were removed and handmade antibiotic loaded cement spacers were implanted. At least three 3 culture samples were collected and histological examinations were also completed to help decide the surgical plan. Vancomycin was the first choice for treating Methicillin–resistant Staphylococcus aureus (MRSA) infected knees when mixing antibiotics. For ensuring not to reduce the fatigue resistance of cement spacer, they choose the utmost amount of antibiotic mixed as 15% of the cement.
Massin et al. (2016)	Not reported
Jenny et al. (2016)	Excision of fistulae when present, careful soft tissue debridement, complete removal of all implants, and careful bone debridement. Multiple bacteriologic samples were taken. The following prostheses were implanted: posterior cruciate ligament preserving, mobile posterostabilized, hypercongruent posterostabilized, semi–constraint posterostabilized, constrained posterostabilized, hinged, and unknown implants. Gentamicin bone cement, plain cement, unknown cement, and uncemented prostheses were used. A muscular flap was performed if required.
Cochran et al. (2016)	Not reported
Zahar A et al. (2016)	Extra–articular debridement of the joint capsule and the synovium was performed. The joint was then opened and debridement performed to remove all infected soft tissue including a complete synovectomy. Collaterals detached and debrided. Solidly fixed implants were explanted with osteotomes or small power saw blades. Intraoperative samples (five for culture, two for histopathology) were taken from the soft tissues and the implant interface, following which intravenous antibiotics specified by an infectious disease consultant were administered. The wound was then lavaged by low–pressure pulsatile lavage with 3000 to 6000 mL of 0.02% poly–hexanid solution. The surgery site was then redraped and gowns, gloves, suction tip, light handles, and instruments were exchanged. Reconstruction of the joint was carried out with implantation of a cemented rotating hinge knee implant. Antibiotic loaded polymethyl–methacrylate (PMMA) bone cement was used for both the fixation of the new implant and reconstruction of bone defects. Therefore, the premixed gentamicin and clindamycin–loaded bone cement was mixed with a maximum of 2 g specific antibiotic powder per 40 g PMMA. Closure achieved and drainage was inserted into the joint for 2 days.
Haddad et al. (2015)	Open aggressive debridement with removal of all components and cement, during which multiple samples are sent to microbiology before administration of antibiotics and the knee is irrigated with hydrogen peroxide and Betadine solutions and pulsatile lavage. The wound is then soaked in aqueous Betadine and the wound edges are approximated. The patient is then redraped, the surgical team rescrubs, and new instruments are used. After a further lavage, implantation of a new prosthesis is per–formed using antibiotic–loaded cement (ALC) according to known sensitivities at a volume of <5% of the total weight of cement powder.
Cury Rde P et al. (2015)	Not reported
Tibrewal et al. (2014)	Knee approached through the old incision, swabs and tissue samples are taken from the joint. All components and cement are removed. Any interface material is taken for both bacteriological and histological examination. The joint is then debrided, excising all tissue of doubtful viability and washed out with copious quantities of normal saline; it is then packed with povidone–iodine-soaked swabs. The wound edges are approximated with sutures and a temporary compressive dressing applied. Appropriate antibiotics, according to the sensitivities established pre–operatively, are administered intravenously and the tourniquet is deflated for 30 min. At this point, the entire operating team re–scrub and put on new gowns and gloves, the patient is re–draped and a new set of instruments is made available. The tourniquet is re–inflated, the knee is reopened and the packs removed. The joint is copiously irrigated again with normal saline and culture swabs are once more taken from bone surfaces. The new components are introduced with antibiotic–impregnated cement, including supplementation with appropriate antibiotics, according to previous cultures and sensitivities. No cement is used around the stems, but the intramedullary canals are dusted with appropriate antibiotic powder. Two suction drains are placed into the joint and the wound is closed.
Klatte et al. (2014)	The mid–vastus approach was used. A minimum of 5 biopsies was taken from around the implants. Intra–operative wound irrigation was performed using pulsatile lavage with polyhexanide prior to implantation of the new prosthesis.
Shanmugasundaram et al. (2014)	Not reported
Baker et al. (2013)	Not reported
Jenny et al. (2013)	Skin incision using the previous scar and approach with tibial tubercle osteotomy if necessary, excision of the fistula when present, careful soft tissue debridement, complete prosthesis removal, and complete bone debridement, including intramedullary reaming. A total of 4 to 8 bacteriologic samples were taken from the debrided tissues and bone. Pulsatile irrigation was used after having completed the debridement. Draping, gloves, and instruments were changed. The reconstruction was performed with standard implants, or posterostabilized implants with stem extension, or a hinged prosthesis owing to ligamentous laxity and substantial bone destruction. All implants were fixed with commercially available gentamicin–loaded cement. Bone defects were filled with allograft or metallic augments according to the surgeon’s preference. A pedicled musculocutaneous flap (medial gastrocnemius or medial soleus muscles) was performed when necessary. Suction drains were left for 48 h according to the surgeon’s preference.
Singer et al. (2012)	Not reported
Whiteside et al. (2011)	Removal of non–absorbable sutures, complete synovectomy; vascularized osteoperiosteal flap osteotomy to expose diaphyseal cement mantles if necessary; and meticulous cement removal using a three–phase debridement starting with rongeurs, followed by curettes, and finishing with high–torque reamer to burr away all surfaces exposed to cement. During debridement, hand–pump irrigation with saline solution of vancomycin (1 g/L), polymyxin (30,000 units/L), and bacitracin (50,000 units/L) was performed repeatedly. After the debridement was finished, the area was cleaned and re–draped, surgical gowns and gloves were changed, and instruments were washed and soaked in the same type of antibiotic solution used for irrigation. Revision total knee implants were inserted using smooth, fluted, press–fit diaphyseal–engaging titanium alloy stems and porous–coated surfaces applied directly to available bone. No cement was used to fix the implants to bone and no bone graft was used to fill defects. A Constavac drain was used for 24–48 h postoperatively.

## Supplementary Materials

The following are available online at http://www.mdpi.com/2077-0383/8/2/174/s1, Table S1: Appendix A—Risk of Bias; Table S2: Appendix B—GRADE Quality.
