# Supplementary material for: Single-Stage Revision Surgery in Infected Total Knee Arthroplasty: A PRISMA Systematic Review
Source: J Clin Med. 2019 Feb 2;8(2):174. doi: 10.3390/jcm8020174 (PMC6406500; doi:10.3390/jcm8020174)
Supplement: Supplementary file 1 [file jcm-08-00174-s001.zip › Supplementary material Search strategy (3).pdf]

Search strategy

String used: (knee) AND (replacement OR arthroplasty) AND ((one-stage exchange) OR (one-stage revision))

Detailed research tab:

("knee"[MeSH Terms] OR "knee"[All Fields] OR "knee joint"[MeSH Terms] OR ("knee"[All Fields] AND "joint"[All Fields]) OR "knee joint"[All Fields]) AND (("replantation"[MeSH Terms] OR "replantation"[All Fields] OR "replacement"[All Fields]) OR ("arthroplasty"[MeSH Terms] OR "arthroplasty"[All Fields])) AND ((one-stage[All Fields] AND ("exchange"[All Fields])) OR (one-stage[All Fields] AND revision[All Fields]))

Databased screened: Pubmed, Science Direct, Embase, Cochrane Library

In addition, we used either key words or MeSH terms. The reference lists of all retrieved articles were reviewed for further identification of potentially relevant studies and assessed using the inclusion and exclusion criteria.
