# Supplementary material for: Single-Stage Revision Surgery in Infected Total Knee Arthroplasty: A PRISMA Systematic Review
Source: J Clin Med. 2019 Feb 2;8(2):174. doi: 10.3390/jcm8020174 (PMC6406500; doi:10.3390/jcm8020174)
Supplement: Supplementary file 1 [file jcm-08-00174-s001.zip › Supplementary tables.pdf]

Appendix A - *Risk Of Bias*

**Study**	**Confounding**	**Selection**	**Intervention Measurment**	**Missing Data**	**Outcome Measurement**	**Reported Results**	**Overall**
Castellani et al.	moderate	Low	moderate	Low	Low	Low	Low
Akgun et al.	moderate	Low	moderate	Low	Low	Low	Low
Ji et al.	moderate	Low	moderate	Low	Low	Low	Low
Li H et al.	moderate	Low	moderate	Low	low	low	low
Massin et al.	moderate	Low	moderate	Low	Low	moderate	moderate
Jenny et al. (2016)	moderate	Low	moderate	Low	low	moderate	moderate
Cochran et al.	low	Low	low	Low	Low	Low	Low
Zahar A et al	moderate	Low	low	Low	Low	Low	Low
Haddad et al.	moderate	Low	moderate	Low	Low	Low	Low
Cury Rde P et al.	moderate	Moderate	Low	moderate	low	low	moderate
Tibrewal et al.	low	low	low	low	low	low	low
Klatte et al.	moderate	low	low	moderate	low	low	low
Shanmugasundaram et al.	low	low	low	low	low	low	low
Baker et al.	low	low	moderate	moderate	low	low	moderate
Jenny et al. (2013)	moderate	low	low	low	low	moderate	moderate
Singer et al.	moderate	low	low	low	low	low	low
Whiteside et al.	moderate	low	low	low	low	low	low

Note: Moderate=the study is sound for a non-randomized study with regard to this domain but cannot be considered comparable to a well-performed randomized trial; Low=the study is comparable to a well-performed randomized trial with regard to this domain

***Appendix B** – GRADE Quality*

Author	Study design	Risk of Bias	Inconsistency	Indirectness	Imprecision	Publication bias	Large effect (RR%)	Plausible confounding	Quality
Castellani et al.	Retrospective Cohort	Low	Not serious	Not serious	serious	Undetected	N/A	Would suggest spurious effect	⊕⊕⊕⊕ High
Ji et al.	Retrospective Case Series	Low	Not serious	Not serious	serious	serious	N/A		⊕⊕⊕ Moderate
Li H et al.	Retrospective Cohort	Low	Not serious	serious	Not serious	Undetected	N/A	No	⊕⊕⊕⊕ High
Massin et al.	Retrospective Cohort	Moderate	Serious	Not serious	Not serious	Undetected	N/A	No	⊕⊕⊕ Moderate
Jenny et al. 2016	Retrospective case-control	Moderate	serious	Not serious	Not serious	Not serious	N/A	No	⊕⊕⊕ Moderate
Cochran et al.	Retrospective Cohort registry	Low	Not serious	Not serious	Not serious	serious	N/A	No	⊕⊕⊕⊕ High

Zahar A et al.	Retrospective Cohort	Low	Not serious	Not serious	serious	Not serious	N/A	No	⊕⊕⊕⊕ High
Haddad et al.	Retrospective Cohort	Low	serious	Not serious	Not serious	Not serious	N/A	No	⊕⊕⊕⊕ High
Cury Rde P et al.	Retrospective Cohort	Moderate	Not serious	Not serious	serious	serious	N/A	No	⊕⊕⊕ Moderate
Tibrewal et al.	Prospective Cohort	low	serious	Not serious	Not serious	Not serious	N/A	No	⊕⊕⊕⊕ High
Klatte et al.	Retrospective Cohort	Low	Not serious	Not serious	serious	serious	N/A	Would reduce the demonstrated effect	⊕⊕⊕ Moderate
Shanmugasundaram et al.	Retrospective Cohort	Low	Not serious	Not serious	serious	serious	N/A	No	⊕⊕⊕ Moderate
Baker et al.	Prospective Cohort	Moderate	Not serious	Not serious	Not serious	Not serious	N/A	No	⊕⊕⊕⊕ High
Jenny et al. (2013)	Observational Cohort Prospective	Moderate	Not serious	Not serious	Not serious	Not serious	N/A	No	⊕⊕⊕⊕ High

Singer et al.	Descriptive Retrospective	Low	Not serious	Not serious	Not serious	Not serious	N/A	No	⊕⊕⊕⊕ High
Whiteside et al.	Retrospective Cohort	Low	Not serious	Not serious	Not serious	Not serious	N/A	No	⊕⊕⊕⊕ High

"Very low"; the true effect is probably markedly different from the estimated effect, "Low"; The true effect might be markedly different from the estimated effect, "Moderate"; the authors believe that the true effect is probably close to the estimated effect, "High"; the authors have a lot of confidence that the true effect is similar to the estimated effect
